# Supplementary material for: Immunomodulatory and Antioxidant Properties of a Novel Potential Probiotic Bacillus clausii CSI08
Source: Microorganisms. 2023 Jan 18;11(2):240. doi: 10.3390/microorganisms11020240 (PMC9962608; doi:10.3390/microorganisms11020240)
Supplement: Supplementary file 1 [file microorganisms-11-00240-s001.zip › Table S1.pdf]

Table S1. Primers used in the study

Primer	Sequence	Product length, bp
gapdh-F	CTTTGACGCTGGGGCTGGCATT	161
gapdh-R	TTGTGCTCTTGCTGGGGCTGGT	
IL-8-F	CAGTTTTGCCAAGGAGTGCT	198
IL-8-R	CAACCCTCTGCACCCAGTTT	
TNF-alpha-F	GCCAGAGGGCTGATTAGAGA	82
TNF-alpha-R	TCTTCTGCCTGCTGCACTT	
IL-17C-F	CAGCTTCTGTGGATAGCGGT	90
IL-17C-R	GAGGTGTTGGAGGCAGACA	
cxcl10-F	TTCAAGGAGTACCTCTCTCTAG	177
cxcl10-R	CTGGATTCAGACATCTCTTCTC	
