# Supplementary material for: Immunomodulatory and Antioxidant Properties of a Novel Potential Probiotic Bacillus clausii CSI08
Source: Microorganisms. 2023 Jan 18;11(2):240. doi: 10.3390/microorganisms11020240 (PMC9962608; doi:10.3390/microorganisms11020240)
Supplement: Supplementary file 1 [file microorganisms-11-00240-s001.zip › Table S2.pdf]

Table S2. Carbohydrate assimilation profile of *B. clausii* CSI08 using API 50 CH system (+ positive; − negative; (+) weakly positive).

Carbohydrate	
Glycerol	+
Erythritol	-
D-Arabinose	-
L-Arabinose	+
D-Ribose	+
D-Xylose	-
L-Xylose	-
D-Adonitol	-
Methyl-β-D-xylopyranoside	-
D-Galactose	-
D-Glucose	+
D-Fructose	+
D-Mannose	+
L-Sorbose	-
L-Rhamnose	+
Dulcitol	+
Inositol	-
D-Mannitol	+
D-Sorbitol	+
Methyl-α-D-mannopyranoside	-
Methyl-α-D-glucopyranoside	-
N-Acetylglucosamine	+
Amygdalin	(+)
Arbutin	-
Esculin Ferric Citrate	+
Salicin	-
D-Cellobiose	+
D-Maltose	-
D-Lactose	-
D-Melibiose	-
D-Saccharose	+
D-Trehalose	+
Inulin	-
D-Melezitose	-
D-Raffinose	(+)
Amidon	(+)
Glycogen	(+)
Xylitol	-

Gentiobiose	-
D-Turanose	-
D-Lyxose	-
D-Tagatose	+
D-Fucose	-
L-Fucose	-
D-Arabitol	-
L-Arabitol	-
Gluconate	-
2-Keto-gluconate	-
5-Ketogluconate	-
