# Supplementary material for: Immunomodulatory and Antioxidant Properties of a Novel Potential Probiotic Bacillus clausii CSI08
Source: Microorganisms. 2023 Jan 18;11(2):240. doi: 10.3390/microorganisms11020240 (PMC9962608; doi:10.3390/microorganisms11020240)
Supplement: Supplementary file 1 [file microorganisms-11-00240-s001.zip › Table S3.pdf]

Table S3. Enzymatic profiles of *B. clausii* CSI08 using the API-ZYM system (+ positive; - negative).

Enzyme	
Esterase (C4:0)	+
Esterase (C8:0)	+
Lipase (C14:0)	-
Leucine arylamidase	-
Valine arylamidase	-
Cystine arylamidase	-
Trypsin	-
α-chymotrypsin	-
Acid phosphatase	-
Alkaline phosphatase	-
Phosphohydrolyase	+
α-Galactosidase	-
β-Galactosidase	+
β-Glucuronidase	-
α-Glucosidase	-
β-Glucosidase	-
β-Glucosaminidase	-
α-Mannosidase	-
α-Fucosidase	-
